# Incorporation of Fiber Bragg Sensors for Shape Memory Polyurethanes Characterization

**DOI:** 10.3390/s17112600

**Published:** 2017-11-11

**Authors:** Nélia Alberto, Maria A. Fonseca, Victor Neto, Rogério Nogueira, Mónica Oliveira, Rui Moreira

**Affiliations:** 1 Instituto de Telecomunicações, Campus Universitário de Santiago, 3810-193 Aveiro, Portugal; rnogueira@av.it.pt; 2 NRG-Research Group, Centre for Mechanical Technology and Automation, Department of Mechanical Engineering, University of Aveiro, 3810-193 Aveiro, Portugal; vneto@ua.pt; 3 GRIDS-Research Group, Centre for Mechanical Technology and Automation, Department of Mechanical Engineering, University of Aveiro, 3810-193 Aveiro, Portugal; mfonseca@ua.pt (M.A.F.); monica.oliveira@ua.pt (M.O.); rmoreira@ua.pt (R.M.)

**Keywords:** fiber Bragg grating sensors, shape memory polymers, polyurethanes, injection molding

## Abstract

Shape memory polyurethanes (SMPUs) are thermally activated shape memory materials, which can be used as actuators or sensors in applications including aerospace, aeronautics, automobiles or the biomedical industry. The accurate characterization of the memory effect of these materials is therefore mandatory for the technology’s success. The shape memory characterization is normally accomplished using mechanical testing coupled with a heat source, where a detailed knowledge of the heat cycle and its influence on the material properties is paramount but difficult to monitor. In this work, fiber Bragg grating (FBG) sensors were embedded into SMPU samples aiming to study and characterize its shape memory effect. The samples were obtained by injection molding, and the entire processing cycle was successfully monitored, providing a process global quality signature. Moreover, the integrity and functionality of the FBG sensors were maintained during and after the embedding process, demonstrating the feasibility of the technology chosen for the purpose envisaged. The results of the shape memory effect characterization demonstrate a good correlation between the reflected FBG peak with the temperature and induced strain, proving that this technology is suitable for this particular application.

## 1. Introduction

Shape memory polymers (SMPs) are a class of smart materials which, in recent years, have been receiving special attention, due to their tremendous technological potential as actuators for biomedical applications, as well as in morphing structures, to be used in aerospace, aeronautic, and marine structural fields [1,2,3]. 

SMPs can be shaped reconfigured and maintained in a transitory shape until receiving a special stimulus, triggering the recovering of the initial and permanent configuration. This trigger effect is usually provided through a thermal stimulus, subjecting the material to a prescribed temperature level. Nevertheless, other alternative activation stimuli can be used, such as light [4,5], magnetic or electric fields [6,7], irradiation and pH changes [8]. 

Moreover, when compared with the conventional shape memory alloys and piezoelectric materials, SMPs have some advantages, mostly concerning their processing capability, lower density and price, and the possibility to actuate when large and distributed deformations are required. 

The experimental characterization of the shape memory effect is usually performed using tensile tests machines with a temperature-controlled chamber [9,10], where different temperature conditions are applied. Other test procedures have also been reported in the literature, which are based on visual and manual techniques. These procedures rely on the analysis of shape fixity and recovery strain of manually bended samples, imposing different bending angles and analyzing the angular displacement during recovery [11]. Those techniques are not sufficiently accurate, especially for low values of shape fixity, recovery strains and reaction times.

The development of improved measurement techniques, towards enhanced levels of accuracy and repeatability is crucial for an efficient and reliable material characterization. Due to their unique characteristics, fiber Bragg grating (FBG)-based systems became one of the most attractive technologies for many sensing applications [12,13,14,15]. Among these features, the immunity to electromagnetic interference, the ability to be used in aggressive environments, the compact size, the high sensitivity, the integration ability into other materials, the multiplexing capability, the absence of electrical current at the measuring point and the real-time measurement possibility can be highlighted. 

This technology was already applied for the characterization of materials. For instance, a work of particular interest reports the use of FBG sensors to monitor the recovery deformation of SMP [16]. In this case, the authors attached the fiber sensors to the SMP sample surface using an adhesive, and demonstrated that the degree and direction (concave/convex) of shape recovery deformation can be easily evaluated. However, since the FBG sensor was glued to the surface of the specimen, a “strain transfer coefficient, *k*” was introduced, which depends on different parameters, such as the geometry of the coating material, the adhesive thickness between the FBG and the SMP, the bond length of the fiber, and the elastic modulus of the materials. Previously, Yin et al. had already glued FBG sensors on a metal sheet of a variable camber wing, which uses a SMP flexible skin, to measure the deflection on some points [17]. Experimental results revealed that the shape of the bending metal sheet can be reconstructed using this sensing technology. 

In both previously reported studies, the sensors were glued to the material, conditioning the strain transfer from the SMP to the Bragg grating. In this work, a different approach is applied: the FBG sensor is integrated inside the material of the specimen, to avoid the abovementioned limitations of the surface solution. To accomplish this goal, and to experimentally assess this approach, a dedicated mold was projected and constructed. Using this special purpose mold, the FBG sensor was fixed and incorporated into the sample during the SMP injection process. This methodology is expected to provide instrumented samples with an improved strain transfer between the material and the sensor, and, consequently, incrementing the measurement accuracy. The experimental setup also allowed the in situ monitoring of the injection process. 

After the incorporation of FBG into the SMP, the samples were tested in order to evaluate its shape memory properties. For that purpose, a special test rig was developed (a customized axial constant load equipment), where a determined load was applied to the sample, and the temperature and deformation were assessed by means of the reflected Bragg wavelength shift. 

## 2. Theory

### 2.1. Shape Memory Programming and Recovery Cycle

The shape memory material used throughout the entire test procedure was a thermo-responsive shape memory polyurethane (SMPU), suitable to be activated using a thermal trigger. To support the shape memory programming procedure, the material was submitted to a complete thermal characterization, in order to obtain its glass transition temperature (T_g_) and melting temperature (T_m_). The latter was then used to attain the transition temperature (T_trans_), which represents the triggering temperature. 

**Figure 1:**
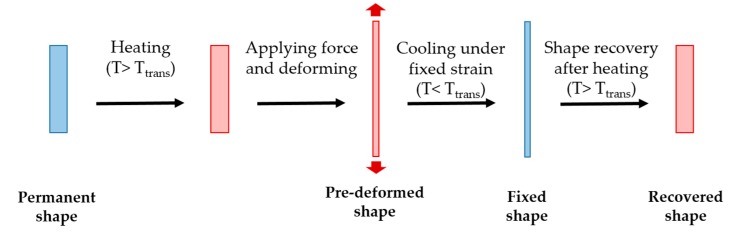
Schematic representation of the basic principle of the shape memory effect in polymers thermally activated.

The shape memory effect of the polyurethane results from its chemical composition, characterized by the hard segment phase (aromatic diisocyanates and small size diols or diamines) and the soft segment phase (aliphatic polyether or polyester diols). As the soft segment has the lower phase transition temperature, it represents the value of the trigger temperature. Above its glass transition temperature (T_trans_ + 20.0 °C), the SMPU can be deformed at any desired shape. This transitory shape can be maintained by cooling the constrained sample below the transition temperature (T_trans_ − 20.0 °C). After this shape programming procedure, the original shape can be recovered by re-heating the SMPU above its transition temperature (T_trans_ + 20.0 °C) [18]. A schematic representation of the shape memory effect in polymers thermally activated is presented in Figure 1.

### 2.2. Fiber Bragg Grating Technology

An FBG is a periodic modulation of the refractive index of the fiber core, typically obtained when an optical fiber is exposed to an ultraviolet radiation pattern. This structure operates as a selective wavelength filter, reflecting only a narrow spectral band with wavelengths that satisfy the Bragg condition (*λ_B_* = 2*n_eff_* Λ ), and transmitting all the others. The effective refractive index (*n_eff_*) and the period (Λ) are affected by changes in strain and temperature, which are manifested by a modification on the center wavelength reflected by the Bragg grating (*λ_B_*), given by Equation (1) [19]:

(1) $$\begin{array}{cc} \Delta \lambda_{B}=\Delta \lambda_{Bl}+\Delta \lambda_{BT} & =2\left(\Lambda \frac{\partial n_{eff}}{\partial l}+n_{eff}\frac{\partial \Lambda }{\partial l}\right)\Delta l+2\left(\Lambda \frac{\partial n_{eff}}{\partial T}+n_{eff}\frac{\partial \Lambda }{\partial T}\right)\Delta T\\ & =S_{l}\Delta l+S_{T}\Delta T \end{array}$$

where Δ*λ_Bl_* is the strain induced wavelength shift and Δ*λ_BT_* is the thermal effect on the same parameter. Parameters *S_l_* and *S_T_* represent the strain and temperature sensitivity coefficients.

## 3. Materials and Methods

### 3.1. SMPU Material

The SMPU used in this work (IROGRAN® A 80 P 4699 L) was supplied by Huntsman HPU Thermoplastic (TPU) and presents a melting temperature ranging from 110.0 to 160.0 °C. The T_trans_, in this case, 50.0 °C, was previously determined through a differential scanning calorimetry (DSC) technique.

### 3.2. Mold Project

**Figure 2:**
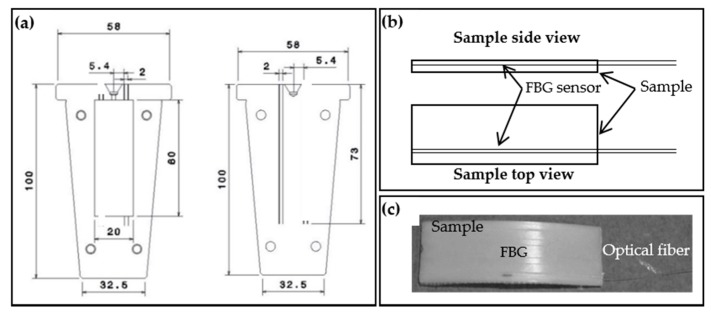
(**a**) Schematic representation of the designed mold tool (dimensions in millimeters); (**b**) Schematic representation of an injected sample with the embedded fiber Bragg grating (FBG) sensor; (**c**) Photograph of a shape memory polyurethane (SMPU) with the embedded FBG.

A dedicated mold, manufactured in stainless steel, was specially designed to allow the simultaneous injection of the SMPU and embedding of the FBG sensors into the samples (Figure 2a). The mold parts were machined to include a v-shaped groove of about 80 µm depth to accommodate the optical fiber sensor inside the SMPU injected sample (Figure 2b,c). It is worth mentioning that the optical fiber used has a diameter of 125 µm, and that the position of the v-shaped groove was defined to ensure the physical integrity of the optical fiber during the injection process. The cylinder movement and the consequent displacement of the melted polymer during the injection process may damage the optical fiber, and consequently becoming the sensor useless. The configuration adopted allows monitoring of the FBG sensor optical signal during the injection process (for quality control), and study of the shape memory effect.

### 3.3. Fiber Bragg Grating Sensors

The FBGs were inscribed into ITU G.652 standard telecommunications fiber, through the phase mask technique, using a KrF excimer pulsed laser emitting at 248 nm. The writing time was around 15 s and the length was 3 mm.

After the inscription process, the gratings were thermally characterized using a climatic chamber (TIRAclima TCC 4034). For that, a heating cycle followed by cooling was imposed, varying from 15.0 to 80.0 °C using step increments of 5.0 °C. For each temperature level, a thermal stabilization period of 15 min was imposed, after which the reflected Bragg wavelength was registered. The interrogation unit operated at a rate of 250 Hz, with a wavelength resolution of 5 pm. For reference, “Free FBG” identifies the fiber Bragg grating before being embedded into the SMPU.

### 3.4. Injection Molding Cycle and Monitoring through FBG Sensors

The optical fibers containing the Bragg sensors were attached to the mold through two fixation points outside of sample cavity, using cyanoacrylate adhesive. These were positioned so as to ensure that the FBGs were in the middle of the SMPU samples (Figure 2c). Before the injection molding procedure, two reflection optical spectra of the FBG were collected: one at the room temperature (RT), 21.2 °C (“FBG @ mold @ 21.2 °C”), with the mold already closed, and another immediately before the material injection (mold inserted into the machine), at a temperature of approximately 70.0 °C (“FBG @ mold @ ~ 70.0 °C”). 

The FBG sensors were embedded into the SMPU samples by injection molding with the following conditions: cylinder temperature (T_cyl_) of 170.0 °C; mold temperature (T_mold_) of 70.0 °C; injection pressure (P_inj_) of 650 bar for 5 s, followed by a pressure (P_pos-inj_) of 500 bar for 2 s. During the SMPU injection into the mold cavity (injection cycle), the reflected Bragg wavelength was monitored. 

**Figure 3:**
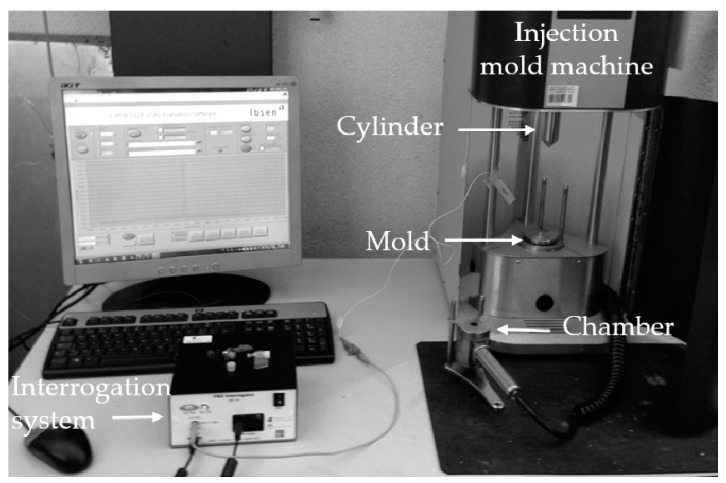
Experimental setup implemented to embed the FBG sensors and monitor the injection cycles.

The samples obtained, shown in Figure 2c, detain a rectangular shape with 60 mm long, 20 mm wide and 2 mm of thickness. The experimental setup is shown in Figure 3. After the injection process, the SMPU with the embedded FBG (identified as “FBG+SMPU” system) were removed from the mold, cooled down to RT (controlled and guaranteed with the thermal chamber) and a new reflected optical spectrum was collected (identified as “FBG+SMPU @ 21.2 °C”). 

### 3.5. Thermal Characterization of the FBG+SMPU System

The FBG+SMPU systems were thermally characterized using similar experimental conditions already applied to calibrate the FBG after the inscription (free FBG). A heating cycle followed by cooling was accomplished, varying from 15.0 to 80.0 °C, using step increments of 5.0 °C. In this case, instead of 15 min, a 30 min stabilization period was applied at each temperature stage. Such stabilization time was estimated from a preliminary test, in which the reflected Bragg wavelength was monitored during the transition between these two temperature values, concluding that this time interval is sufficient for the whole system to reach the thermal equilibrium.

### 3.6. Shape Memory Characterization

**Figure 4:**
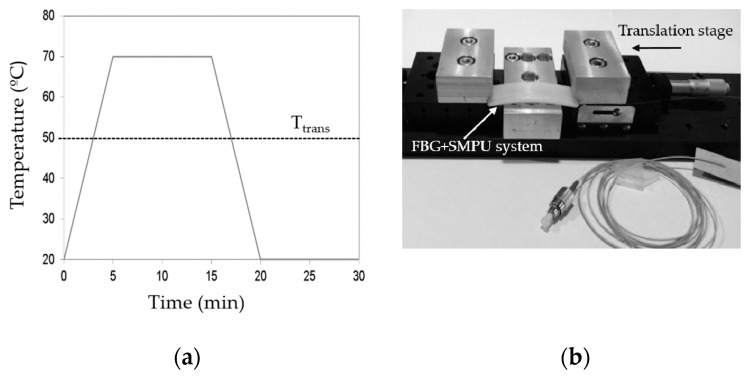
(**a**) Applied shape memory cycle; (**b**) Experimental setup implemented to apply the axial strain to the SMPU.

In order to analyze the shape memory effect of the SMPU, the test samples were subjected to a programming cycle followed by a restoring stage, shown in Figure 4a. Figure 4b presents the experimental apparatus developed to apply a constant axial load to the samples, using a micrometer screw to impose a pre-determined and controlled displacement. Several reflection optical spectra were recorded during the applied cycle (shape memory programming cycle and restoring period). Moreover, all measurements undertaken were carried out inside the temperature controlled chamber.

## 4. Results and Discussion

### 4.1. Monitoring of the Injection Cycle

An FBG was embedded into the SMPU during the injection molding of the material, and the cycle was also monitored taking advantage of the FBG being inside the mold. Furthermore, in what concerns the injection molding cycle monitoring, it should be said that variables such as injection pressure, time and temperature were also assessed globally. 

**Figure 5:**
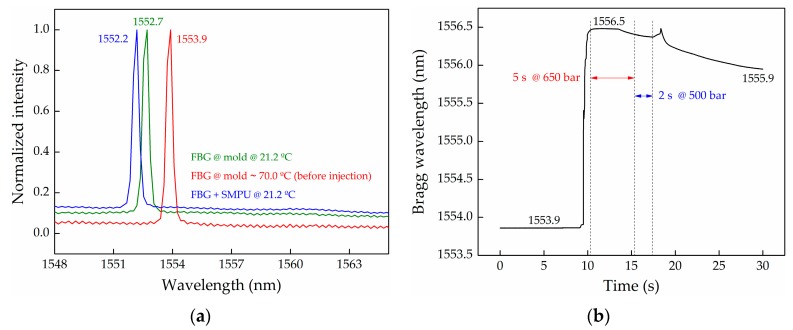
(**a**) FBG reflection optical spectra at different stages of the injection process; (**b**) Evolution of the Bragg wavelength along with the injection time of the SMPU into the mold cavity.

Figure 5a presents the reflection optical spectra of the FBG sensor at three different stages: FBG sensor attached to the mold, and outside of the injection machine, at 21.2 °C (“FBG @ mold @ 21.2 °C”); FBG sensor attached to the mold, inside the injection machine, at ~ 70.0 °C and before injection of the material (“FBG @ mold @ ~ 70.0 °C (before injection)”); and after removing the FBG+SMPU system from the mold cavity, at 21.2 °C (“FBG+SMPU @ 21.2 °C”). 

**Figure 6:**
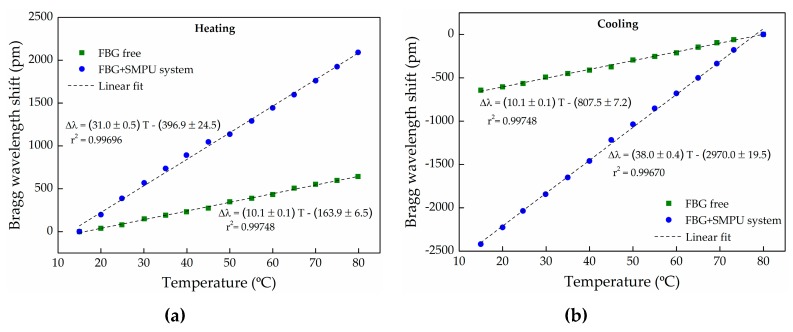
Thermal characterization of the free and the FBG+SMPU system during (**a**) heating and (**b**) cooling.

From the first two spectra, a red-shift of the FBG reflection peak of 1.2 nm (1552.7 to 1553.9 nm) was observed. Such a result is not consistent with the temperature increase from 21.2 to 70.0 °C. According to the thermal sensitivity coefficient of 10.1 pm/°C, determined for this free FBG after the Bragg grating inscription process (the results are presented in Figure 6), a temperature increase of about 118.8 °C would be expected to explain this wavelength shift. This discrepancy may result from the difference between the temperature value predicted and used in this calculation (70.0 °C) and the real mold temperature, which may be higher, added to the mold + FBG system manipulation during its positioning inside the chamber. The FBG accommodated into the mold cavity may move from its initial position, originating an FBG peak shift. However, this difference is not expected to interfere with the analysis described hereafter.

Immediately after the injection process finished, the mold was removed from the injection machine, cooled down, and the FBG+SMPU system was extracted from the mold. The Bragg wavelength of the FBG+SMPU system was measured at 21.2 °C, and at this stage, a decrease of 0.5 nm was observed, compared with the Bragg wavelength registered before the injection, for the same temperature. This reading may reflect the effect of the polymer post-molding contraction during the cooling stage from the melting temperature (~170.0 °C) to the room temperature.

The results presented in Figure 5b describe the evolution of the reflected Bragg wavelength during the injection cycle of the SMPU inside the mold cavity. The overall acquisition time was set as 30 s, starting a few seconds before the injection cycle starts, and extending few seconds after the completion of the cycle. A red-wavelength shift of the reflected Bragg wavelength (2.6 nm) was registered, being consistent with an FBG distension movement and the injection of the SMPU into the mold at 650 bar. Such strain effect is superimposed with thermal variations, since the SMPU was injected at 170.0 °C, and the mold cavity was initially at ~70.0 °C. However, with the implement sensing scheme, the strain and temperature effects cannot be dissociated. Thus, this real-time injection cycle monitoring can only provide a global quality signature of the entire process, and not a precise quantitative indication on the strain and temperature variations. 

Over the first 5 s of the injection cycle, two distinct behaviors can be observed: a plateau at 1556.5 nm where the strain and temperature effects could be balanced; and a small decreasing of the Bragg wavelength mainly due to the temperature effect. In the following 2 s at 500 bar (compaction stage), the trend of the Bragg wavelength is identical, however in this case the temperature effect is superimposed with the reduction of the pressure. At the end of the cycle (7 s), without any applied pressure, the graph shows a sharp increase of the reflected Bragg wavelength followed by a decrease, denoting the removal of the applied pressure, superimposed with a continuous decrease of the temperature inside the mold cavity. 

### 4.2. Thermal Characterization of the FBG+SMPU System

The results of thermal characterization for both free FBG (conducted before the injection) and FBG+SMPU system (accomplished after embedding the FBG into the SMPU) are presented in Figure 6. In the heating cycle case, the free FBG and the FBG+SMPU system Bragg wavelengths, obtained at 15.0 °C, were used as reference. In the cooling cycle case, those values were the ones registered at 80.0 °C. 

The stabilization period considered between the two temperature stages was different for both samples: 15 min for the free FBG, and 30 min for the FBG+SMPU system. This difference is justified by the fact that, in the last case, the FBG sensor was embedded into the SMPU, and therefore it requires more time to reach the thermal equilibrium. Note that this is a thermoplastic polymer with low thermal conductivity (0.18 W m^−1^ K^−1^) and a small thermal diffusivity (5 × 10^−7^ m^2^/s).

A significant difference on the thermal sensitivities in the heating cycle was observed: 10.1 pm/°C for the free FBG, and 31.0 pm/°C for the FBG+SMPU system. The latter is almost three times higher, evidencing the increase of the thermal sensitivity of the FBG, when embedded inside the polymer matrix. This behavior results from the high thermal expansion coefficient of the SMPUs (around 10^−4^ °C^−1^), compared with the ~0.55 × 10^−6^ °C^−1^ for the case of the silica optical fiber [19]. As the temperature increases, the SMPU expands, stretching the Bragg grating, and consequently, a greatest variation of the Bragg wavelength is obtained, compared with the shift registered for a free FBG under the same conditions. The same effect was previously observed by Li et al. [20].

From the same graphical representation, it can also be observed that, for the FBG+SMPU system, the sensitivities are slightly different during the heating and the cooling cycles (the sensitivity is 38.0 pm/°C during the cooling). This behavior, observed in successive heating/cooling cycles (data not displayed here), may be related with the thermomechanical properties of the SMPU, however, in the future, this issue should be carefully investigated and understood. This effect does not occur for the free FBG, since the thermal sensitivities coefficients are identical for both heating and the cooling (10.1 pm/°C) cycles. 

### 4.3. Shape Memory Cycle and Recovery

**Figure 7:**
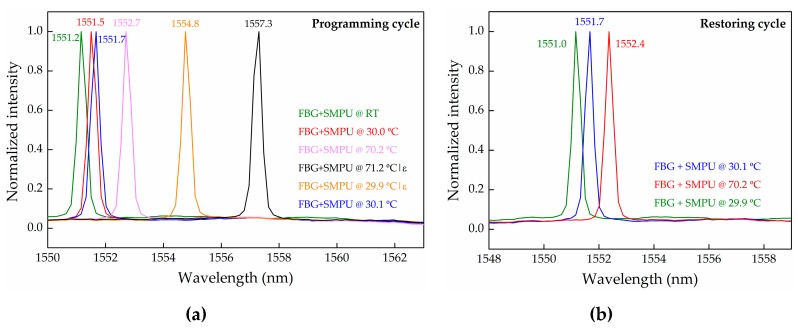
Reflection optical spectra of the FBG+SMPU system along with the (**a**) programming and the (**b**) restoring cycle.

Figure 7a shows several reflection optical spectra for the FBG+SMPU system at different temperatures and levels of the applied load (consequently, different values of imposed deformation), obtained with the experimental setup described in Figure 4b.

With a null displacement (no load condition), a red-wavelength shift of 1.5 nm in the reflected Bragg wavelength was registered when the temperature increased from 21.2 to 70.2 °C. This result agrees with the one calculated through the thermal sensitivity coefficient. 

After the application of a predetermined load level, corresponding to a displacement of 4.5 mm on the longitudinal axis, an increase on the Bragg wavelength of 4.6 nm is observed (from 1552.7 nm @ 70.2 °C without load, to 1557.3 nm @ 71.2 °C with load), which results from the imposed strain. Note that the wavelength shift (31.0 pm) due to the temperature variation from 70.2 to 71.2 °C is neglected. 

When the temperature is decreased to T_trans_ − 20.0 °C (29.9 °C in this case), in order to crystalize the soft segments and the temporary shape, a blue-wavelength shift of 2.5 nm is detected, much higher than the expected wavelength shift of 1.6 nm due to the temperature change. It is believed that the unexpected shift of 0.9 nm is related to the FBG relaxation process occurring inside the polyurethane matrix. Such a relaxation effect may be associated to the accommodation of the optical fiber within the soft polymeric chains, releasing some strain initially induced in the FBG sensor. After removing the FBG+SMPU system from the experimental setup, a shape relaxation of the sample was observed, clearly revealed by the blue-wavelength shift of the Bragg grating, from 1554.8 to 1551.7 nm (−3.1 nm). It is worth mentioning that the increase of the Bragg wavelength caused by the temperature variation from 29.9 to 30.1 °C is negligible. However, the FBG+SMPU system kept a transitory shape, since a Bragg wavelength variation of 0.6 nm is still maintained. This value results from the 0.9 nm and 3.1 nm (values above discussed) subtraction to the 4.6 nm registered when the sample was submitted to a determined load level, corresponding to a displacement of 4.5 mm. Considering the Bragg wavelength regaining analysis, this result allows us to estimate a 87% shape recovery, from the applied shape memory.

The results obtained during the restoring cycle can be depicted from Figure 7b. When the FBG+SMPU system was re-heated from temperature 30.1 to 70.2 °C, a red-wavelength shift from 1551.7 to 1552.4 nm (0.7 nm) was observed. From the thermal characterization, the expected Bragg wavelength shift due to the thermal effect is 1.2 nm, which is higher than the observed value of 0.7 nm. The difference of 0.5 nm is attributed to the SMPU strain relaxation, meaning that the SMPU is restoring its original shape. Again, the wavelength shift results from the superposition of two effects: temperature and strain, which seems to be consistent with the polymer contraction during the shape recovery period. However, when cooling down the system to 29.9 °C, a shift of 1.4 nm is observed, a value smaller than the expected 1.5 nm (due to temperature). This effect may be explained by the fact that the system did not recover its total original shape, keeping some induced strain. Since this work is intended to represent essentially a proof-of-concept, results from only one programming and restoring cycle are presented. Nonetheless, later it may be interesting to assess the hysteresis phenomenon when the SMPU sample is submitted to a larger number of repeated programming and recovering cycles. Some studies point out a decrease of the shape recovery with the increase of the cycle number.

Future work will be devoted to solving the strain and temperature cross-sensitivity. Some schemes that may allow this shortcoming to be overcome have already been proposed in the literature. For instance, the inscription of two superimposed FBGs in the same position of the fiber optic, providing simultaneous measurement of longitudinal and transversal strain and temperature [21]. Recently, and with a similar purpose, Pereira et al. embedded two FBG sensors with a certain angle between them in polymeric tensile test specimens [22]. Nonetheless, it should be mentioned that the latter methodology, however interesting, will entail a new mold adjustment and a new accommodation process for the Bragg sensors, since the polymeric samples are obtained by distinct processing methods, in our case through the injection molding. Although in this first experiment only one sensor was embedded into the SMPU, a multi-point shape memory monitoring scheme is intended to be implemented soon, using FBG arrays. In these conditions, several other influencing parameters should be addressed, such as the embedded FBGs position dependence on the strain/curvature. In the thermal characterization case, the sensor response is not expected to be dependent on the FBGs position into the sample.

Nevertheless, the main aim of this work was successfully attained; to establish and demonstrate the suitability and adequacy of the embedding method for the application envisaged, since it was paramount to guarantee the FBG sensors integrity and functionality during the entire procedure, namely sensors accommodation and samples processing.

## 5. Conclusions

In this work, FBGs inscribed in optical fibers were successfully embedded into SMPU samples. The latter was accomplished by processing polymer samples through injection molding, allowing the real-time monitoring of the entire process. The thermal sensitivities for a free FBG and the FBG+SMPU system were determined, and the results clearly show that the incorporation of the FBG into the SMPU treble its thermal sensitivity. With the experimental setup developed, it was possible to apply a constant and controlled load to the FBG+SMPU system, and the reflected FBG wavelength was acquired. For the shape memory programming cycle a Bragg wavelength shifted due to the applied load, and temperature changes was observed. However, these two effects are superimposed and at this investigation stage it is not possible to completely dissociate both effects. After the shape recovery cycle, the SMPU kept some strain, not completely acquiring its original shape; however, a blue-wavelength shift was observed corresponding to strain relaxation (due to restoring of the initial shape). This work has shown the ability to incorporate FBGs in polymers during the injection process, which can be used not only to characterize smart materials, but also to access further details on other engineering polymers.
